# Anticancer Activity of Continentalic Acid in B-Cell Lymphoma

**DOI:** 10.3390/molecules26226845

**Published:** 2021-11-12

**Authors:** Byeol-Eun Jeon, Chan-Seong Kwon, Ji-Eun Lee, Keumok Moon, Jaeho Cha, Inmyoung Park, Sara Koh, Myunghee Yoon, Sang-Woo Kim, Jeong Nam Kim

**Affiliations:** 1 Department of Integrated Biological Science, Pusan National University, Busan 46241, Korea; starsilver20@naver.com (B.-E.J.); ckstjd5091@naver.com (C.-S.K.); dlwldms4535@naver.com (J.-E.L.); moonko81@nate.com (K.M.); jhcha@pusan.ac.kr (J.C.); 2 Department of Microbiology, Pusan National University, Busan 46241, Korea; 3 Department of Asian Food and Culinary Arts, Youngsan University, Busan 48015, Korea; inmpark@gmail.com; 4 Department of Biological Sciences, Southern Methodist University, Dallas, TX 75206, USA; kohs@mail.smu.edu; 5 Division of Hepatobiliary and Pancreas Surgery, Department of Surgery, Biomedical Research Institute, Pusan National University, Busan 46241, Korea; ymh@pusan.ac.kr; 6 Department of Biological Sciences, Pusan National University, Busan 46241, Korea

**Keywords:** *Aralia continentalis*, diterpenes, B-cell lymphoma, anti-cancer activities

## Abstract

*Aralia continentalis* has been used in Korea as a folk remedy for arthralgia, rheumatism, and inflammation. However, its anti-lymphoma effect remains uncharacterized. Here, we demonstrate that *A. continentalis* extract and its three diterpenes efficiently kill B-lymphoma cells. Our in vitro and in vivo results suggest that the cytotoxic activities of continentalic acid, a major diterpene from *A. continentalis* extract, are specific towards cancer cells while leaving normal murine cells and tissues unharmed. Mechanistically, continentalic acid represses the expression of pro-survival Bcl-2 family members, such as Mcl-1 and Bcl-xL. It dissociates the mitochondrial membrane potential, leading to the stimulation of effector caspase 3/7 activities and, ultimately, cell death. Intriguingly, this agent therapeutically synergizes with roflumilast, a pan-PDE4 inhibitor that has been successfully repurposed for the treatment of aggressive B-cell malignancies in recent clinical tests. Our findings unveiled that *A. continentalis* extract and three of the plant’s diterpenes exhibit anti-cancer activities. We also demonstrate the synergistic inhibitory effect of continentalic acid on the survival of B-lymphoma cells when combined with roflumilast. Taken in conjunction, continentalic acid may hold significant potential for the treatment of B-cell lymphoma.

## 1. Introduction

Lymphoma is a blood cancer that develops from B lymphocytes, T lymphocytes, and natural killer cells. It is categorized into Hodgkin’s lymphoma (HL) (10% of cases) and non-Hodgkin’s lymphoma (NHL) (90% of cases). Most NHLs are derived from B-cells. B-cell NHLs include follicular lymphoma (FL), mantle cell lymphoma (MCL), diffuse large B cell lymphoma (DLBCL), and Burkitt’s lymphoma (BL) [1]. The prominent features of BL include rapid proliferation and c-Myc deregulation, which is caused by the translocation of the c-Myc gene on chromosome 8 to the immunoglobulin heavy or light chain loci [2,3]. DLBCL is the most common subtype of B-cell NHLs, accounting for approximately 30% of newly diagnosed cases in the United States. Gene expression profiling studies have suggested that DLBCL can be divided into germinal center B cell-like (GCB) and activated B cell-like (ABC) DLBCL, with the latter associated with poor survival compared with the former [4,5]. Genetic aberrations that characterize GCB DLBCL are Bcl-2 translocations that lead to the inhibition of apoptosis [6]. A hallmark of ABC DLBCL is the constitutive activation of nuclear factor kappa-light-chain-enhancer of activated B cells (NF-kB) signaling. The inhibition of this signaling promotes apoptosis, indicating that ABC DLBCL depends on NF-kB for survival [5,7,8]. In line with this, activating mutations signaling molecules in the NF-kB pathway, such as MyD88, CARD11, and B-cell-receptor components, CD79A and CD79B, are frequently found in ABC DLBCL tumor biopsies [7,8].

Cyclophosphamide, doxorubicin, vincristine, and prednisone (CHOP) therapy is widely used as a standard treatment for DLBCL patients. Addition of rituximab (Rituxan, Genentech/Biogen Idec), a chimeric monoclonal antibody targeting CD20 on B-cells, to CHOP therapy improved survival rates without significantly increasing toxicity and is considered the most important advancement in the treatment of B-cell lymphoma [9]. Rituximab can induce B-cell destruction through several mechanisms, including complement activation, antibody-dependent cellular cytotoxicity (ADCC) [10,11], and apoptosis induced by caspase-3 activation [12]. Despite rituximab and other advancements in B-cell lymphoma treatment, around 40% of DLBCL patients still die from the disease. In this regard, studies on the disease and continual efforts for novel drug development remain imperative.

Approximately 60% of anticancer drugs currently used have been derived from natural products with the majority attributed to plants. Many reports have extensively shown that extracts from *Aralia continentalis* exert antibacterial, anti-inflammatory, antidepressant, antidiabetic, and antioxidant potentials. [13,14,15]. In particular, our group has previously demonstrated that the hexane layer of *A. continentalis* extracts shows substantial antibacterial activity to the carcinogenic pathogen *Streptococcus mutans* [16]. Furthermore, three diterpenoid acids (ent-kaur-16-en-19-oic acid (kaurenoic acid), *epi*-continentalic acid, and continentalic acid) were mainly identified from the hexane fraction and exhibit similar levels of antibacterial activity as displayed in the ongoing study (unpublished data). In addition to its antibacterial activity, two diterpenes (kaurenoic and continentalic acids) also exert cytotoxic effects. Cytotoxicity of these diterpenes against various tumor cell lines including human breast cancer, colon cancer, and leukemia has been evaluated using MTT assays [17,18,19]. It has been reported that the classes of kaurene diterpene and their derivatives present anti-tumor activity via the apoptosis-inducing properties via the activation of apoptotic genes (e.g., *fas*, *caspase-8*, and *caspase-3*) [20,21]. Such properties were also observed in HepG2 cells exposed to continentalic acid. It has also been demonstrated that apoptosis of HepG2 cells exposed to this active compound in a dose-dependent manner is induced by the activation of caspase-3, Bak, and Bax with the downregulation of Bcl-2 [19]. However, the anti-lymphoma effects of these diterpenes have not been tested.

Although molecular and cellular characterization of B-cell lymphoma have contributed to the improvement of survival rates of patients with the disease, one-third of patients still relapse, necessitating continued effort for developing novel therapeutics. In the present study, we sought to investigate the potential anti-cancer effect of *A. continentalis* extract and its three diterpenes on B lymphoma cells. Our data demonstrate that they efficiently promoted apoptosis of B lymphoma cells with different cells-of-origin and various genetic abnormalities, suggesting that they have a tumor-suppressive effect in a broad range of B-cell lymphoma. Importantly, continentalic acid, a major diterpene from *A. continentalis*, has a very low cytotoxic effect on normal cells in vitro and in vivo; normal murine splenocytes and bone marrow cells were exposed to continentalic acid in vitro, and the murine lung, kidney, heart, and liver tissues were analyzed after mice were treated with this agent, which demonstrated minimal toxicity. Additionally, continentalic acid could synergize with roflumilast, a pan-phosphodiesterase (PDE) 4 inhibitor, to enhance the pro-apoptotic effect on B lymphoma cells. Roflumilast has been successfully tested for the treatment of hematological cancers in several clinical trials. When combined, the apoptosis-inducing effect was much greater than single agents alone. Given the almost inevitable drug resistance during chemotherapy, our results suggest that resistance to roflumilast could be overcome by combining with continentalic acid. These results suggest that continentalic acid could be a novel therapeutic for the treatment of patients with B-cell lymphoma.

## 2. Results

### 2.1. Aralia continentalis Extract Induces Apoptosis of Human Lymphoma Cell Line

**Figure 1:**
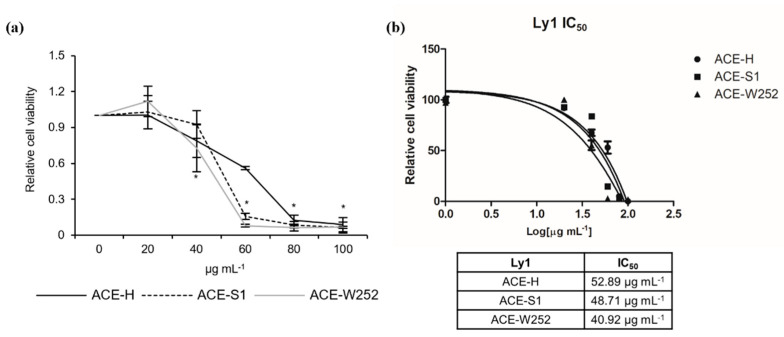
ACE-H, ACE-S1, and ACE-W252 reduces cell viability in Ly1 human B lymphoma cells. (**a**) Ly1 cells were treated with ACE-H, ACE-S1, or ACE-W252 (0, 20, 40, 60, 80, and 100 μg mL^−1^) for 24 h, and cell viability was measured using an MTS assay. (**b**) The IC50 values of ACE-H, ACE-S1, and ACE-W252 in Ly1 cells were calculated using GraphPad Prism 5 software (GraphPad Software, Inc., San Diego, CA, USA). Statistical significance was calculated using the two-tailed Mann–Whitney test (* *p* < 0.05).

To investigate the potential anti-lymphoma effect of *A. continentalis* extracts, Ly1 human B lymphoma cells were exposed to hexane fractions (ACE-H), silica gel fractions (ACE-S1), or W252 column fractions (ACE-W252) (Figure S1), followed by measurement of cell viability using MTS assays. As shown in Figure 1a, these fractions efficiently reduced the viability of Ly1 cells in 24 h. Analysis of IC_50_ (half-maximal inhibitory concentration) values of ACE-H, ACE-S1, and ACE-W252 fractions of *A. continentalis* extracts yielded 52.89, 48.71, and 40.92 μg mL^−1^, respectively (Figure 1b). Given that IC_50_ values are lowered in fractions from later purification steps, we presume that compounds with potential anti-cancer effect are becoming more concentrated as the extracts go through purification process.

### 2.2. Three Diterpenes from A. continentalis Extracts Induce Apoptosis in Human Lymphoma Cell Lines

**Figure 2:**
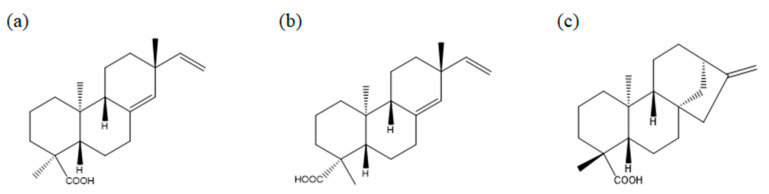
Identification of three diterpenes from the extract of *A. continentalis*. A chemical formula for (**a**) *epi*-continentalic acid (*ent*-pimara-8(14),15-dien-18-oic acid), (**b**) continentalic acid (*ent*-pimara-8(14),15-dien-19-oic acid), and (**c**) kaurenoic acid (*ent*-kaura-16-en-19-oic acid) is C_20_H_30_O_2_, C_20_H_30_O_2_, and C_20_H_30_O_2_, respectively.

**Figure 3:**
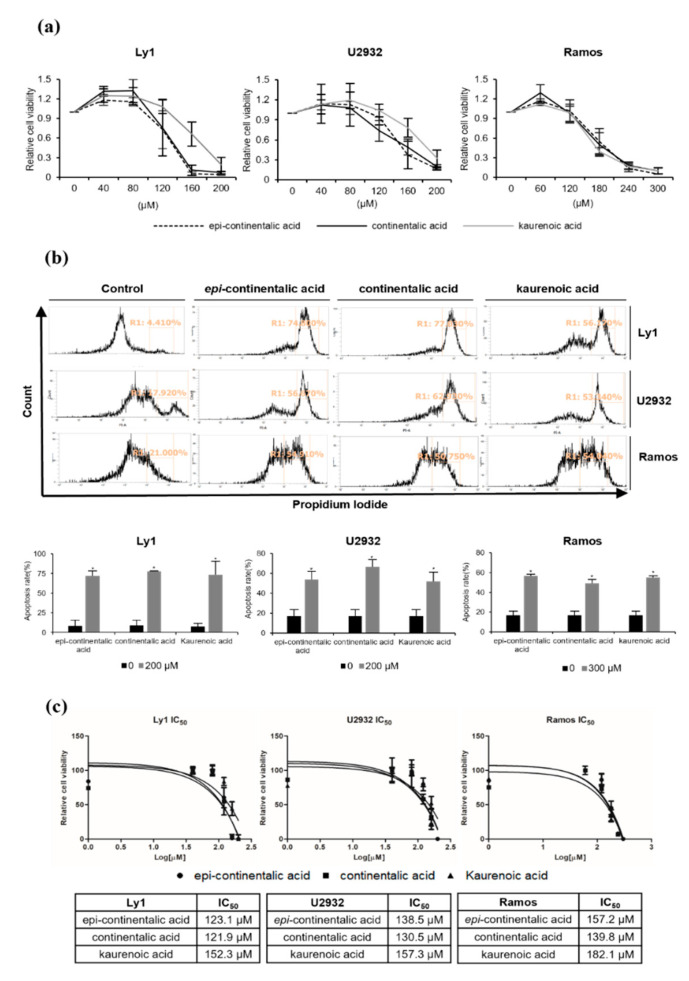
*Epi*-continentalic acid, continentalic acid, and kaurenoic acid induce apoptosis in human lymphoma cell lines. (**a**) Cell viability of Ly1, U2932, and Ramos was measured by MTS assay after treatment with *epi*-continentalic acid, continentalic acid, or kaurenoic acid for 24 h, as indicated. (**b**) Relevant cells were exposed to *epi*-continentalic acid, continenetalic acid, or kaurenoic acid for 24 h, as indicated, and the apoptotic rate was measured by PI staining followed by FACS analysis. The two-tailed Mann–Whitney test was used to calculate statistical significance (* *p* < 0.05). (**c**) The IC_50_ values of *epi*-continentalic acid, continentalic acid, and kaurenoic acid in Ly1, U2932, and Ramos cells were calculated using GraphPad Prism 5 software.

Next, we used the octadecyl–silica (ODS) reverse-phase high-performance liquid chromatography (HPLC) column to isolate single compounds responsible for anticancer activity in *A. continentalis*, and three diterpenes, *epi*-continentalic acid, continentalic acid, and kaurenoic acid, were identified (Figure 2). The viability of three human B lymphoma cells with diverse cell-of-origins, Ly1 (GCB DLBCL), U2932 (ABC DLBCL), and Ramos (BL), was examined after treating with increasing concentrations of these agents, leading to the remarkable suppression of viability in a dose-dependent manner in all three cell lines (Figure 3a). Consistently, apoptotic fractions measured by propidium iodide (PI) staining and flow cytometry (FACS) analysis in these cells were significantly increased upon exposure to the compounds (Figure 3b). To obtain a better insight into the anticancer effects, IC_50_ was analyzed, which ranged from 121.9 to 182.1 μM. Ramos cells were the most resistant, while Ly1 cells were the most sensitive (Figure 3c). Of the three components, continentalic acid had the lowest IC_50_ values of 121.9, 130.5, and 139.8 μM in Ly1, U2932, and Ramos, respectively (Figure 3c). It is unclear whether sensitivity to continentalic acid is related to the origins of these cells, warranting further study. We compared the efficacy of continentalic acid with that of doxorubicin, a well-established chemotherapeutic drug called an anthracycline. Human B lymphoma cell lines Ly1 and Ramos were exposed to increasing concentrations of doxorubicin, resulting in a decrease of cell viability in a dose-dependent manner (Figure S2a). The IC_50_ values of doxorubicin in Ly1 and Ramos cells were 1.084 μM and 125.4 nM, respectively, suggesting that this agent is very efficient in killing B lymphoma cells as compared with continentalic acid (Figure S2b).

### 2.3. Three Dipertenes from A. continentalis Extracts Have Minimal Toxicity towards Normal Cells

**Figure 4:**
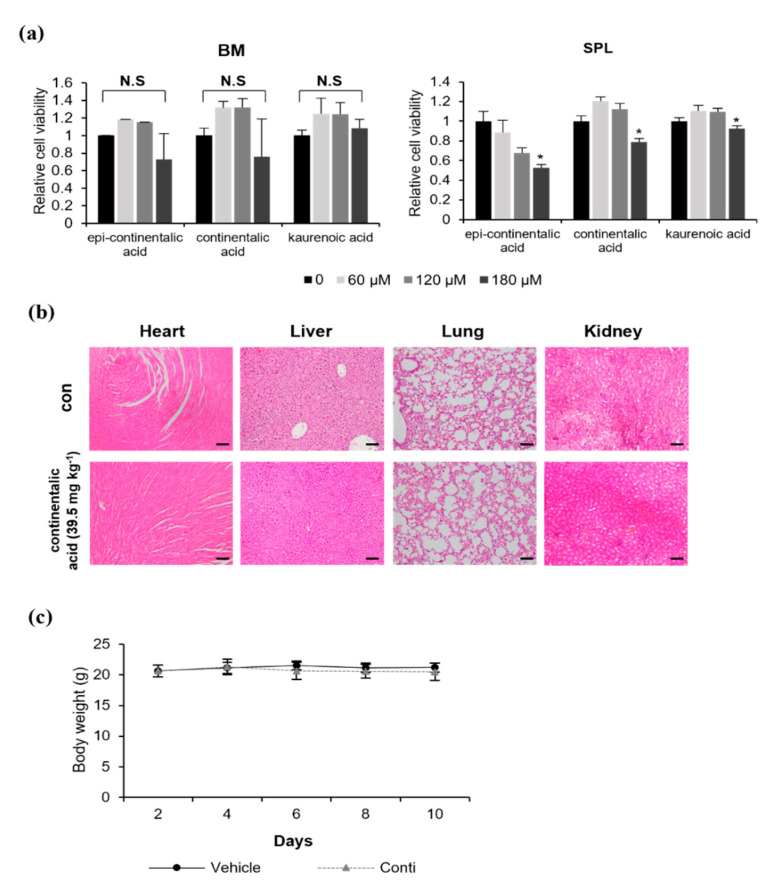
*Epi*-continentalic acid, continentalic acid, and kaurenoic acid exhibit low toxicity towards normal cells in vitro and in vivo. (**a**) Bone marrow cells (BM) and splenocytes (SPL) isolated from wild-type C57BL/6 mice were treated with *epi*-continentalic acid, continentalic acid, and kaurenoic acid (0, 60, 120, and 180 µM) for 24 h, followed by assessment of cell viability by MTS assays. Statistical significance was calculated using the two-tailed one-way analysis of variance (ANOVA) test (* *p* < 0.05). (**b**) A vehicle (50 μL of DMSO) or continentalic acid (130 µM) was injected into athymic nude mice (BALB/c-nu, 4 weeks old, male, *n* = 5 per treatment group) intraperitoneally daily for 10 days. The hearts, livers, lungs, and kidneys removed from the mice were fixed with 4% paraformaldehyde and embedded in paraffin. For histological analysis, tissue sections were stained with hematoxylin and eosin and observed at 100× magnification under the microscope. Scale bar, 100 µm. (**c**) Mouse body weights were measured every 2 days for 10 days during the treatment.

To investigate whether the cell-death-inducing activity of these compounds is specific to cancer cells, we analyzed their toxicity towards normal cells in vitro and in vivo. Normal murine bone marrow cells and splenocytes were treated with increasing doses of three diterpenes, followed by measurement of cell viability by MTS assays. These compounds did not affect the survival of bone marrow cells, while the viability of splenocytes was significantly reduced by 10~50% at 180 µM (Figure 4a). Based on these data and the results in Figure 3a, we concluded that continentalic acid effectively killed B lymphoma cells with minimal toxicity towards normal cells in vitro and decided to further characterize continentalic acid. To examine impact on normal cells in vivo, a vehicle (50 µL of dimethyl sulfoxide, DMSO) or continentalic acid (39.5 mg kg^−1^) was injected into athymic nude mice intraperitoneally daily for 10 days, and heart, liver, lung, and kidney tissue sections were hematoxylin and eosin (H&E)-stained, followed by observation under the microscope (Figure 4b). We did not find any abnormality in tissue morphology upon treatment with continentalic acid compared with mice in the control group. Additionally, body weights in the vehicle and treatment group did not show any difference, suggesting no systemic toxicity (Figure 4c). As stated, a previous study has shown that HepG2, a liver hepatocellular carcinoma cell line, can be killed by continentalic acid, and our results indicate that this agent has minimal or no effect on normal liver cells/tissues, which suggests that continentalic acid can induce apoptosis in liver cancer cells, leaving normal liver cells/tissues unharmed. In conjunction, these results suggest that the anticancer activity of three diterpenes, especially continentailic acid, is specific towards B lymphoma cells.

### 2.4. Continentalic Acid Induces Apoptosis Depending on Caspase and Mitochondria by Regulating Bcel-2 Family Members

**Figure 5:**
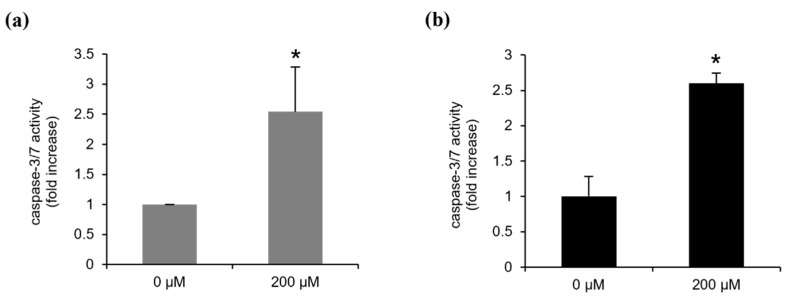
Continentalic acid inhibits caspase 3/7 activity. Caspase-3/7 activity was monitored using ELISA-based bioluminescence assays after treatment with vehicle (0 μM of continentalic acid; 1% DMSO) or continentalic acid (200 μM) for 24 h in (**a**) Ly1 and (**b**) U2932. Statistical significance was calculated using the two-tailed one-way analysis of variance (ANOVA) test (* *p* < 0.05).

**Figure 6:**
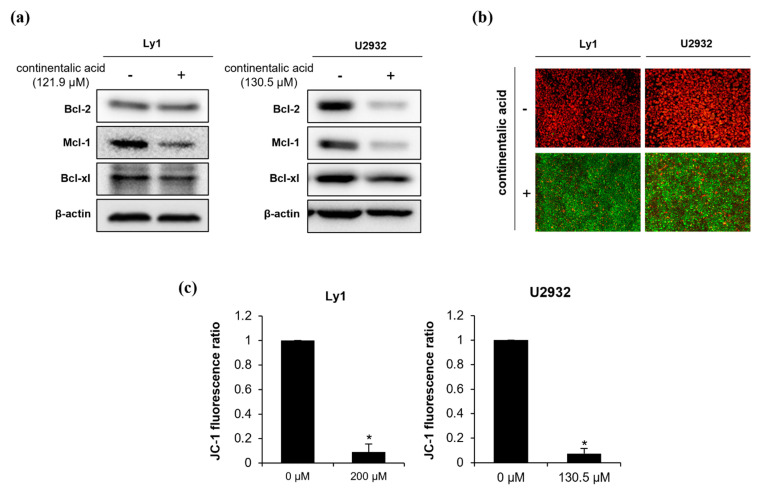
Continentalic acid reduces expression of anti-apoptotic Bcl-2 family members and dissociates mitochondrial membrane potential (MMP). (**a**) Continentalic acid was added in Ly1 and U2932 cells, and the expression of anti-apoptotic Bcl-2 family members Bcl-2, Mcl-1, and Bcl-xl was detected by Western blot 24 h later. β-actin served as a control for equal loading. A representative of three independent experiments is shown. (**b**) Ly1 and U2932 were treated with continentalic acid for 24 h and stained with JC-1, followed by monitoring of MMP by fluorescence microscopy. Red (J-aggregates) and green (J-monomers) colors represent intact and disrupted MMP, respectively. (**c**) MMP was calculated using flow cytometry to monitor the JC-1 fluorescence ratio of J-aggregates (red) and J-monomers (green). Statistical significance was calculated using the two-tailed Mann–Whitney test (* *p* < 0.05).

Caspases are a family of cysteine-aspartic acid-specific proteases that regulate apoptosis. Their activation is the result of the apoptosis signaling pathway. They are categorized into initiator (caspase-3 and -7) and effector caspases (caspase-8, -9, and -10). We investigated whether continentalic acid induces cell death via caspase activation (Figure 5). Ly1 and U2932 B lymphoma cells were treated with 200 μM continentalic acid for 24 h, which increased caspase-3/7 activity by about 2.5-fold compared with vehicle treatment. These results suggest that continentalic acid may stimulate caspase activity to induce apoptosis. Intrinsic and extrinsic apoptosis pathways converge on the regulation of activity/levels of pro- and anti-apoptotic Bcl-2 family members. This disrupts mitochondria membrane potential (MMP) by altering the permeability of the mitochondrial membrane, which leads to cytochrome C release and activation of caspases. To determine the possible involvement of Bcl-2 family members and mitochondria in cell death triggered by continentalic acid, Ly1 and U2932 cells were exposed to this agent and levels of anti-apoptotic Bcl-2 family members, such as Bcl-2, Mcl-1, and Bcl-xL (Figure 6a). Overall, U2932 was more sensitive regarding the repression of protein levels. Interestingly, Bcl-2 protein levels were downregulated in U2932, but not in Ly1, while Mcl-1 and Bcl-xL levels were significantly repressed in both cell lines, suggesting that continentalic acid may kill B lymphoma cells by downmodulation of Mcl-1 and Bcl-xL, while Bcl-2 may play a minor role in this process. We subsequently tested if the suppression of pro-survival Bcl-2 family members might affect MMP. MMP was monitored by fluorescence microscopy and flow cytometry following the exposure of Ly1 and U2932 cells to continentalic acid for 24 h. JC-1 fluorescence ratio indicates the complete dissipation of MMP by this agent (Figure 6b,c). Together, these results suggest that continentalic acid disrupts MMP and increases caspase activity by reducing the expression of anti-apoptotic Bcl-2 family members.

**Figure 7:**
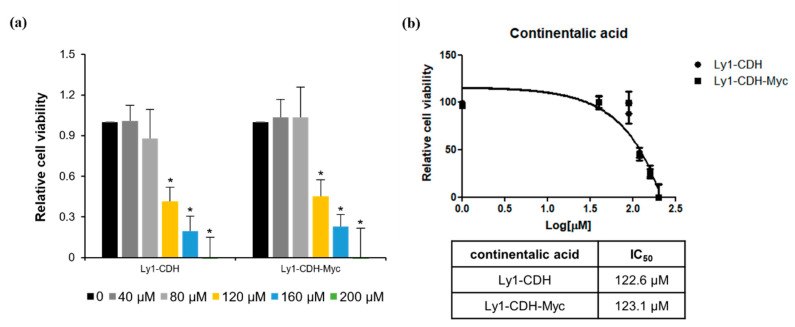
Continentalic acid inhibits cell survival Myc-independently. (**a**) Ly1-CDH and Ly1-CDH-Myc cells were exposed to increasing concentrations of continentalic acid for 24 h, followed by measurement of cell viability by MTS assays. Statistical significance was calculated using the two-tailed one-way ANOVA test (* *p* < 0.05). (**b**) The IC_50_ values of continentalic acid in Ly1-CDH and Ly1-CDH-Myc cells were calculated using GraphPad Prism 5 software, which shows virtually no difference between Ly1-CDH and Ly1-CDH-Myc cells.

Myc dysregulation is one of the most common genetic abnormalities in B-cell lymphoma. In addition, this oncogene has been shown to play a critical role in the survival and aggressiveness of hematological cancers. To directly test whether continentalic acid-induced cell death is associated with regulation of Myc, we used Ly1 cells ectopically expressing Myc (Ly1 CDH-Myc) and control cells transduced with a control lentiviral vector (Ly1 CDH) that were well-characterized in previous studies [22]. We presumed that Myc overexpression would render the cells resistant to continentalic acid if Myc was involved in this process. Ly1 CDH and CDH-Myc cells exhibited the same pattern of susceptibility to this agent (Figure 7a); the IC_50_ values of the former and the latter are 122.6 and 123.1 µM, respectively (Figure 7b). This result demonstrates that the continentalic-acid-induced cell-killing effect is independent of Myc.

### 2.5. Synergy between Roflumilast and Continentalic Acid

**Figure 8:**
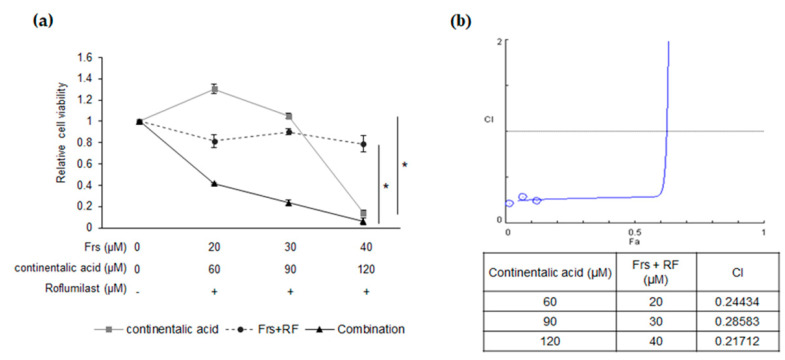
Continentalic acid therapeutically synergizes with forskolin/roflumilast to induce apoptosis in DLBCL cells. (**a**) Ly1 was treated with forskolin (0 to 50 μM) or continentalic acid (0 to 150 μM) or both in the absence or presence of Roflumilast (40 μM), and cell viability was performed by MTS assay after 24 h. Statistical significance was calculated using the two-tailed one-way ANOVA test (* *p* < 0.05) (**b**) Combination index (CI) values were calculated by CompuSyn software to determine the synergistic effect of Roflumilast and continentalic acid on Ly1 cell viability by comparing combination and single-drug treatments. Synergy levels are as follows: <0.1, very strong synergism; 0.1–0.3, strong synergism; 0.3–0.7, synergism; 0.7–0.85, moderate synergism; 0.85–0.90, slight synergism; 0.90–1.10, nearly additive; 1.10–1.20, slight antagonism; 1.20–1.45, moderate antagonism; and 1.45–3.30, antagonism.

Roflumilast (Daxas) is an anti-inflammatory drug that acts as a selective and potent inhibitor of phosphodiesterase-4 (PDE-4). The drug is approved by the European Union and FDA in the United States for the treatment of chronic obstructive pulmonary disease (COPD). PDE4B, an isoform of PDE4, is highly expressed in refractory DLBCL patients and the anticancer effect of PDE4 inhibitors has been confirmed in B-cell lymphoma in vitro and in vivo, associated with repression of the SYK/AKT/mTOR pathway. Furthermore, roflumilast has been successfully repurposed in recent small clinical trials for the treatment of DLBCL. These data suggest that this drug may have a beneficial effect on patients with this disease. Given that Mcl-1 is a major downstream target of the SYK/AKT/mTOR signaling pathway and that continentalic acid significantly represses Mcl-1 levels, we hypothesized that combinatory treatment of these two agents may have a synergistic inhibitory effect on the survival of B lymphoma cells. The addition of either roflumilast up to 90 µM or continentalic acid in Ly1 cells had either a modest or no effect on cell survival, whereas co-treatment of these agents resulted in remarkable suppression of cell viability (Figure 8a). To quantify the putative synergistic effect of combinatorial treatment, we calculated combination index (CI) values using the CompuSyn software (ver. 1.0) (ComboSyn, Inc., Paramus, MJ, USA) based on the MTS assay data in Figure 8a. The results show substantial synergism when these agents are co-treated (Figure 8b).

## 3. Discussion

*A. continentalis* is a medicinal herb traditionally used in Korea for the treatment of arthralgia, rheumatism, lumbago, and lameness. *A. continentalis* extracts and three diterpenes exhibited anti-lymphoma activities by suppressing cell viability and enhancing apoptosis in B lymphoma cell lines. The mechanism underlying this effect is associated with activation of the caspase cascade, which is caused by the suppression of anti-apoptotic Bcl-2 family members and the dissipation of MMP. Intriguingly, roflumilast and continentalic acid cooperated to induce cell killing in B-cell lymphoma. The inhibition of Mcl-1 may play an important role in the interaction between these two agents.

Our study shows that continentalic acid, a diterpene from *A. continentalis* extracts, induces apoptosis via inhibition of pro-survival Bcl-2 family members, especially Mcl-1 and Bcl-xL. Presently, it is unclear how the expression of these genes is inhibited by continentalic acid. Myc is found to be frequently dysregulated in B-cell lymphoma, and the gene is amplified in Ly1 GCB DLBCL cells. Previous studies suggested that Myc is one of the upstream regulators. However, given our data demonstrating that continentalic acid triggers apoptosis via the Myc-independent pathway, Mcl-1 and Bcl-xL levels may not be regulated by Myc. Investigation on the mechanism by which continentalic acid regulates the expression of these genes is necessary.

Roflumilast, a pan-PDE4 inhibitor, has been successfully tested in recent clinical trials as a potential treatment option for B-cell hematologic malignancies. When this agent was administered with prednisone, 66% of evaluable patients with advanced B-cell malignancies exhibited a partial response or stable disease with low toxicity. The most common side effects were fatigue, anorexia, and neutropenia in some patients. In another recent clinical study involving patients with relapsed/refractory DLBCL, roflumilast was combined with cytarabine; although the difference was not statistically significant, the patient group receiving roflumilast and ESHAP (etoposide, cisplatin, methylprednisolone, and cytarabine) showed better rates of complete response (CR), overall response rate (ORR), and 1-year progression-free survival (PFS) as compared with the patient group receiving ESHAP only. Our data suggest that continentalic acid has no or minimal toxicity towards normal cells in vitro and in vivo, and roflumilast could be combined with continentalic acid to treat B-cell lymphoma patients. Mechanistically, the combinatorial effects of these two agents appear to be associated with the amelioration of Mcl-1 levels.

Mcl-1 is an important antiapoptotic gene, promoting cell survival in various hematological malignancies, including multiple myeloma (MM), acute myeloid leukemia (AML), and NHL [23,24,25]. Aberrant expression of Mcl-1 is associated with chemoresistance, and the efficacy of Mcl-1 inhibitors, such as S63845 (MIK665), AMG176, and AZD5991, is being tested in clinical trials [26,27]. PDE4B is one of the genes that are highly expressed in refractory DLBCL patients, and one of the main downstream targets of roflumilast is Mcl-1, indicating that this agent may be useful in overcoming chemoresistance by repressing Mcl-1 levels. Intriguingly, Mcl-1 was the most sensitive to continentalic acid in both Ly1 and U2932 B lymphoma cells, suggesting that this gene may play a critical role in continentalic acid-induced apoptosis. Given that roflumilast efficiently inhibits expression of Mcl-1, synergistic suppression of cell viability by combinatorial treatment of roflumilast and continentalic acid may be explained, at least in part, by the efficacious inhibition of Mcl-1. Together, the synergistic anticancer activity of these agents may converge on the downmodulation of Mcl-1 levels.

Given high IC_50_ values of continentalic acid in B lymphoma cells, it is imperative to improve the efficacy of the agent to be able to be used clinically, which could be achieved by modifications of chemical moieties of continentalic acid. For example, mycophenolic acid (MPA), an IMP-dehydrogenase (IMPDH) inhibitor, is being tested in clinical trials for the treatment of advanced multiple myeloma. Hydroxamic acid analogues (MAHAs) are synthetic compounds that replace the carboxyl group of MPA with a hydroxamic acid moiety, resulting in a more potent antiproliferative agent than the parent compound [28]. Additionally, a recent study demonstrated that a nanopharmaceutical system using a TAT-enhanced cell/tissue penetration strategy efficiently inhibited tumor growth while significantly reducing adverse cytotoxicity in vivo [29]. Therefore, we believe that our future studies should be directed toward enhancing the efficacy of continentalic acid with minimal side effects.

## 4. Materials and Methods

### 4.1. Cell Culture and Antibodies

Human lymphoma cell lines (Ly1, Ramos, and U2932) were cultured in RPMI-1640 medium (Hyclone, USA) supplemented with 10% fetal bovine serum (FBS; Hyclone, Melbourne, VIC, Australia), 1% L-glutamine, 1% *N*-2-hydrocyethylpiperazine-*N*’-2-ethanesulfonic acid (HEPES) buffer, and 1% penicillin/streptomycin at 37 ℃ in a 5% CO_2_ incubator. Normal cells (splenocytes and bone marrow cells) from wild-type C57BL/6JJmsSlc mice were cultured in a DMEM medium with 10% FBS (Hyclone).

The primary antibodies against Bcl-2 (Santa Cruz Biotechnology; sc-7381), Mcl-1 (Santa Cruz Biotechnology; sc-819), Bcl-xL (eBioscience; 14-6994-81), and β-actin (Santa Cruz Biotechnology; sc-4778) were used for western blots. HRP-conjugated anti-rabbit and anti-mouse secondary antibodies were from Bethyl Laboratories, Inc. (A120-101p and A90-116p-33) (Montgomery, TX, USA).

### 4.2. Preparation of Aralia continentalis Extracts and Isolation of Single Compounds

The dried roots of *Aralia continentalis* were purchased in a local market in Seoul, Korea, in May 2020. The overall process of extraction was summarized in Supplementary Figure S1. The roots were ground using a food processor. A total of 50 g of the roots were extracted in 1 L of ethanol by sonication at 50 °C for 1 h, and the supernatant was collected [30]. This process was repeated in triplicate, and all supernatants were mixed and filtered (No. 2, Toyo Roshi Kaisha, Ltd., Japan). The supernatant was concentrated with a rotary vacuum evaporator (Eyela, Japan) under reduced pressure at 50 °C. The ethanol extract (ACE, 2.58 g) was dissolved in 10% ethanol to avoid aggregation of the extract and fractionated with *n*-hexane and water [31]. The *n*-hexane fraction (ACE-H, 1.12 g) was concentrated and subjected to silica gel column chromatography (particle size: 0.063–0.200 mm, 6 cm × 40 cm, Merck, Darmstadt, Germany) and eluted with *n*-hexane/ethyl acetate (5:1, *v*/*v*). Fractions (ACE-S1, 0.34 g) containing diterpenoids were separated by preparative liquid chromatography (prep LC) equipped with an ultraviolet detector (JAI Co., Tokyo, Japan) at room temperature. The ACE-S1 was loaded onto a JAIGEL-W252 gel filtration column (2 × 50 cm, JAI Co.) in the prep LC and eluted with methanol. The peak was observed at 205 nm. The collected fractions (ACE-W252, 0.18 g) containing diterpenoids were concentrated with a rotary vacuum evaporator and loaded onto an ODS column (2 cm × 25 cm, YMC Co., Kyoto, Japan) with methanol/acetonitrile/0.1% trifluoroacetic acid (42.75:52.25:5, *v*/*v*/*v*) to obtain *epi*-continentalic acid (12 mg), continentalic acid (54 mg), and kaurenoic acid (30 mg). The fractions from each chromatography were analyzed by thin layer chromatography (TLC) as described previously [32] with some modifications. Each fraction was spotted directly onto TLC silica gel 60 plates (Merck, Darmstadt, Germany). The plate was chromatographed with a developing solution containing *n*-hexane/ethyl acetate (5:1, *v*/*v*) and visualized by immersion in a solution containing 1% (*w*/*v*) *N*-(1-naphthyl)-ethylenediamine and 20% (*v*/*v*) sulfuric acid in methanol after heating at 110 °C for 10 min.

### 4.3. Cell Viability and Apoptosis Assays

To measure cell viability, the CellTiter 96 AQueous One Solution [3-(4,5-dimethylthiazol-2-yl)-5-(3-carboxymethoxyphenyl)-2-(4-sulfophenyl)-2H-tetrazolium] cell proliferation assays (MTSs) were conducted according to the manufacturer’s instructions (Promega, Madison, WI, USA) after treatment with ACE-H, ACE-S1, ACE-W252, *epi*-continentalic acid, continentalic acid, and kaurenoic acid alone or in combination with roflumilast as indicated [22]. Briefly, 3 × 10^4^ cells per well of 96-well plates were seeded in RPMI1640 with 10% FBS, and the cells were treated for 24 h as indicated, followed by measurement of cell viability by MTS assays. To test the synergistic effect of roflumilast and continentalic acid, 3 × 10^5^ cells per well of 12-well plates were seeded in RPMI1640 with 5% FBS, and the cells were treated for 48 h as indicated, followed by measurement of cell viability by MTS assays.

To analyze apoptotic rates, human lymphoma cell lines were plated in 12-well plates at a density of 1 × 10^6^ cells per well and treated with *epi*-continental acid, continental acid, and kaurenoic acids (0, 200, or 300 μM) for 24 h. The apoptosis rate was analyzed using flow cytometry (FACSVerse, BD biosciences) after staining with Propidium Iodide (BD biosciences, Franklin Lakes, NJ, USA).

### 4.4. Analysis of Caspase 3/7 Activity and Mitochondrial Membrane Potential

To confirm caspase 3/7 activity in human lymphoma cell lines, Ly1 and U2932 cells were exposed to continentalic acid 200 μM for 24 h. Caspase-Glo 3/7 Assay reagent (G8090; Promega) was added, and cells were incubated 1 h at RT, followed by luminescence measurement using GloMax^TM^ Microplate multi-mode reader (Promega) [33].

The mitochondrial membrane potential (MMP) was analyzed using fluorescence microscopy and FACS after treatment of human lymphoma cell lines (Ly1 or U2932) with continentalic acid (200 or 130.5 μM) for 24 h, followed by incubation with JC-1 (5,5′,6,6′-tetrachloro-1,1′,3,3′-tetraethylbenzimidazolylcarbocyanine iodide; Abnova, Taipei, Taiwan) dye for 15 min at 37 ℃.

### 4.5. Western Blot Analysis

To perform western blot, human lymphoma cell lines (Ly1 or U2932) were seeded in a 12-well plate at a density of 1 × 10^6^ per well, and continentalic acid was added as indicated. After 24 h, cells were harvested and lysed in RIPA buffer (ELPIS Biotechnology; Daejeon, Korea) with 1 mM Na-vanadate, 50 mM β-glycerophosphate disodium salt, β-mercaptoethanol (142 mM; BioWORLD, Visalia, CA, USA), ProteaseArrest^TM^ (G-Bioscience; Maryland Heights, MO, USA), and EDTA (5 mM; G-Bioscience). Protein samples were boiled in a 1X sample buffer at 100 °C for 10 min and loaded onto the polyacrylamide gels. After gel electrophoresis, proteins were transferred onto Immobilon-P transfer membranes, followed by blocking in 1% bovine serum albumin (BSA; MP Biomedicals, Santa Ana, CA, USA) dissolved in Tris-buffered saline containing 0.1% Tween-20 (TBST). Membranes were incubated in primary antibodies for 16 h at 4 °C on a rotor and each washed three times for 5 min with TBST. The membrane was incubated with anti-rabbit or anti-mouse secondary antibodies at RT for 1 h and washed three times for 10 min each. Protein bands on the membrane were exposed to a chemiluminescent substrate (EzWestLumi plus (ATTO, Osaka, Japan)) and visualized using the Luminograph Ⅱ Imaging system (ATTO Technology, Osaka, Japan) [34].

### 4.6. Animal Studies

To study the toxicity of the *epi*-continentalic acid, continentalic acid, and kaurenoic acid in vitro, splenocytes and bone marrow cells were extracted from wild-type C57BL/6 mice (9 weeks old male) and exposed to these diterpenes as indicated, followed by the MTS assays. For in vivo toxicity testing, athymic nude mice (BALB/c-nu; 5 weeks old male; Central Lab. Animal Inc., Seoul, Korea) were divided into two groups (*n* = 5 per group), and vehicle (DMSO) or continentalic acid (3.93 mg kg^−1^) was injected intraperitoneally for 10 days, followed by histological analysis of the lung, heart, kidney, and liver. The body weights of mice were measured every other day for 10 days. Animal experiments conducted in this study were reviewed and approved by the Pusan National University-Institutional Animal Care and Use Committee (PNU-2021-3056).

### 4.7. Hematoxylin and Eosin (H&E) Staining

H&E staining was performed as previously described [35]. The tissues from mice were fixed with a 10% neutral buffered formalin solution (Sigma; St Louis, MO, USA) and embedded in paraffin. Paraffin sample blocks were sectioned into 4 μM, deparaffinized, hydrated, and stained with H&E. H&E-stained samples were observed with an Olympus CX31 microscope (Olympus Corporation, Tokyo, Japan) at 100× magnification. Representative images were captured using Images Plus 2.0 software (Motic Co. Ltd., Xiamen, China).

### 4.8. Statistical Analysis

All experiments were repeated at least three times independently. Data are presented as mean ± standard deviation (SD). Statistically significant differences were calculated by a Mann–Whitney U test and one-way analysis of variance (ANOVA) test with Tukey’s post hoc test using the Microsoft Office Excel and GraphPad Prism software (GraphPad Software, Inc., San Diego, CA, USA).

## 5. Conclusions

We characterized the anticancer effects of three dipterpenes, *epi*-continentalic acid, continentalic acid, and kaurenoic acid, from *A. continentalis* extracts using B lymphoma cells. Continentalic acid was effective in triggering cell death with the lowest IC_50_. The agent reduced the expression of anti-apoptotic Bcl-2 family members, disrupted MMP, and stimulated effector caspase 3/7 activities, which ultimately leads to apoptosis. Importantly, the cell-killing effect of continentalic acid was specific towards B lymphoma cells, leaving normal murine cells unaffected in vivo and in vitro. This suggests that the agent may have minimal adverse effects when applied to patients with B-cell malignancies. It is worth noting that roflumilast synergizes with continentalic acid to suppress the survival of B lymphoma cells, which possibly associates with inhibition of Mcl-1. Further mechanistic insights into the interaction between roflumilast and continentalic acid may offer an opportunity to improve the efficacy of these agents in the treatment of patients with B-cell lymphoma.

## Data Availability

Not applicable.

## Supplementary Materials

The following are available online: Figure S1: Extraction process of diterpenoids from the roots of *Aralia continentalis*, Figure S2: The anti-lymphoma effect of doxorubicin.

## Sample Availability

Samples of the compounds are available from the authors.

## References

[1] Armitage J.O., Gascoyne R.D., Lunning M.A., Cavalli F.J.T.L. (2017). Non-hodgkin lymphoma. Lancet.

[2] Rabbitts T., Hamlyn P.H., Baer R. (1983). Altered nucleotide sequences of a translocated c-myc gene in Burkitt lymphoma. Nature.

[3] Dalla-Favera R., Bregni M., Erikson J., Patterson D., Gallo R.C., Croce C.M. (1982). Human c-myc onc gene is located on the region of chromosome 8 that is translocated in Burkitt lymphoma cells. Proc. Natl. Acad. Sci. USA.

[4] Li S., Young K.H., Medeiros L.J.J.P. (2018). Diffuse large B-cell lymphoma. Diagn. Histopathol..

[5] Alizadeh A.A., Eisen M.B., Davis R.E., Ma C., Lossos I.S., Rosenwald A., Boldrick J.C., Sabet H., Tran T., Yu X. (2000). Distinct types of diffuse large B-cell lymphoma identified by gene expression profiling. Nature.

[6] Schuetz J.M., Johnson N.A., Morin R.D., Scott D.W., Tan K., Ben-Nierah S., Boyle M., Slack G.W., Marra M.A., Connors J.M. (2011). BCL2 mutations in diffuse large B-cell lymphoma. Leukemia.

[7] Ngo V.N., Young R.M., Schmitz R., Jhavar S., Xiao W., Lim K.-H., Kohlhammer H., Xu W., Yang Y., Zhao H. (2011). Oncogenically active MYD88 mutations in human lymphoma. Nature.

[8] Lenz G., Davis R.E., Ngo V.N., Lam L., George T.C., Wright G.W., Dave S.S., Zhao H., Xu W., Rosenwald A. (2008). Oncogenic CARD11 mutations in human diffuse large B cell lymphoma. Science.

[9] Coiffier B., Lepage E., Brière J., Herbrecht R., Tilly H., Bouabdallah R., Morel P., Van Den Neste E., Salles G., Gaulard P. (2002). CHOP chemotherapy plus rituximab compared with CHOP alone in elderly patients with diffuse large-B-cell lymphoma. N. Engl. J. Med..

[10] Weng W.-K., Levy R. (2003). Two immunoglobulin G fragment C receptor polymorphisms independently predict response to rituximab in patients with follicular lymphoma. J. Clin. Oncol..

[11] Reff M.E., Carner K., Chambers K.S., Chinn P.C., Leonard J.E., Raab R., Newman R.A., Hanna N., Anderson D.R. (1994). Depletion of B cells in vivo by a chimeric mouse human monoclonal antibody to CD20. Blood.

[12] Weiner G.J. (2010). Rituximab: Mechanism of action. Seminars in Hematology.

[13] Lee B., Hong R., Lim P., Cho D., Yeom M., Lee S., Kang K.S., Lee S.C., Shim I., Lee H. (2019). The ethanolic extract of *Aralia continentalis* ameliorates cognitive deficits via modifications of BDNF expression and anti-inflammatory effects in a rat model of post-traumatic stress disorder. BMC Complement. Altern. Med..

[14] Liaquat I., Khan A.-U., Khan S. (2021). Pharmacological evaluation of continentalic acid for antidiabetic potential. Biomed. Pharmacother..

[15] Yang D.K., Lee S.-J., Adam G.O., Kim S.-J. (2020). *Aralia continentalis kitagawa* extract attenuates the fatigue induced by exhaustive exercise through inhibition of oxidative stress. Antioxidants.

[16] Lee Y.-C., Cho S.-G., Kim S.-W., Kim J.N. (2019). Anticariogenic potential of Korean native plant extracts against *Streptococcus mutans*. Planta Medica.

[17] Mongelli E., Pomilio A.B., Sánchez J.B., Guerra F.M., Massanet G.M. (2002). ent-Kaur-16-en-19-oic acid, a KB cells cytotoxic diterpenoid from *Elaeoselinum foetidum*. Phytother. Res..

[18] Arora B., Sharma E., Agrawal S., Agrawal M. (2015). In vitro cytotoxicity of methanol extract from aerial parts of *Aralia cachemirica* and purified continentalic acid. Indian J. Pharm. Sci..

[19] Kwon T.O., Jeong S.-I., Kwon J.W., Kim Y.C., Jang S.I. (2008). Continentalic acid from *Aralia continentalis* induces growth inhibition and apoptosis in HepG2 cells. Arch. Pharmacal Res..

[20] Lizarte Neto F.S., Tirapelli D.P., Ambrosio S.R., Tirapelli C.R., Oliveira F.M., Novais P.C., Peria F.M., Oliveira H.F., Carlotti Junior C.G., Tirapelli L.F. (2013). Kaurene diterpene induces apoptosis in U87 human malignant glioblastoma cells by suppression of anti-apoptotic signals and activation of cysteine proteases. Braz. J. Med. Biol. Res..

[21] Hueso-Falcon I., Giron N., Velasco P., Amaro-Luis J.M., Ravelo A.G., de las Heras B., Hortelano S., Estevez-Braun A. (2010). Synthesis and induction of apoptosis signaling pathway of ent-kaurane derivatives. Bioorg. Med. Chem..

[22] Nam J., Kim D.U., Kim E., Kwak B., Ko M.J., Oh A.-Y., Park B.-J., Kim Y.W., Kim A., Sun H. (2019). Disruption of the Myc-PDE4B regulatory circuitry impairs B-cell lymphoma survival. Leukemia.

[23] Wuillemetoumi S., Robillard N., Gomez P., Moreau P., le Gouill S., Avet-Loiseau H., Harousseau J.-L., Amiot M., Bataille R. (2005). Mcl-1 is overexpressed in multiple myeloma and associated with relapse and shorter survival. Leukemia.

[24] Craig R.W. (2002). MCL1 provides a window on the role of the BCL2 family in cell proliferation, differentiation and tumorigenesis. Leukemia.

[25] Glaser S.P., Lee E., Trounson E., Bouillet P., Wei A., Fairlie W., Izon D.J., Zuber J., Rappaport A.R., Herold M. (2012). Anti-apoptotic Mcl-1 is essential for the development and sustained growth of acute myeloid leukemia. Genes Dev..

[26] Xiang W., Yang C.-Y., Bai L. (2018). MCL-1 inhibition in cancer treatment. OncoTargets Ther..

[27] Hird A.W., Tron A.E. (2019). Recent advances in the development of Mcl-1 inhibitors for cancer therapy. Pharmacol. Ther..

[28] Chen L., Wilson D., Jayaram H.N., Pankiewicz K.W. (2007). Dual inhibitors of inosine monophosphate dehydrogenase and histone deacetylases for cancer treatment. J. Med. Chem..

[29] Liu J., Zhao Y., Guo Q., Wang Z., Wang H., Yang Y., Huang Y. (2012). TAT-modified nanosilver for combating multidrug-resistant cancer. Biomaterials.

[30] National Institute of Food and Drug Safety Evaluation (2018). Araliae Continentalis Radix.

[31] Moon K., Cha J. (2020). Enhancement of antioxidant and antibacterial activities of *Salvia miltiorrhiza* roots fermented with *Aspergillus oryzae*. Foods.

[32] Moon K., Lee S., Park H., Cha A.J. (2021). Enzymatic synthesis of resveratrol α-glucoside by amylosucrase of *Deinococcus geothermalis*. J. Microbiol. Biotechnol..

[33] Kim E., Nam J., Chang W., Zulfugarov I.S., Okhlopkova Z., Olennikov D., Chirikova N.K., Kim S.-W. (2018). Angelica gigas nakai and decursin downregulate myc expression to promote cell death in b-cell lymphoma. Sci. Rep..

[34] Shim H., Nam J., Kim S.-W.J.G. (2020). NF-κB p65 represses microRNA-124 transcription in diffuse large B-cell lymphoma. Genes Genomics..

[35] Kim J., Kim J.N., Park I., Sivtseva S., Okhlopkova Z., Zulfugarov I.S., Kim S.-W. (2020). *Dracocephalum palmatum* Stephan extract induces caspase-and mitochondria-dependent apoptosis via Myc inhibition in diffuse large B cell lymphoma. Oncol. Rep..

