# Supplementary material for: Anticancer Activity of Continentalic Acid in B-Cell Lymphoma
Source: Molecules. 2021 Nov 12;26(22):6845. doi: 10.3390/molecules26226845 (PMC8625780; doi:10.3390/molecules26226845)
Supplement: Supplementary file 1 [file molecules-26-06845-s001.zip › molecules-1378289-supplementary.pdf]

Supplementary Materials

Anticancer Activity of Continentalic Acid in B-Cell Lymphoma

Byeol-Eun Jeon[1], Chan-Seong Kwon[1], Ji-Eun Lee[1], Keumok Moon[2], Jaeho Cha[1,2], Inmyoung Park[4], Sara Koh[5], Myunghee Yoon[6], Sang-Woo Kim[1,3*] and Jeong Nam Kim[1,2*]

[1] Department of Integrated Biological Science, Pusan National University, Busan 46241, Republic of Korea; starsilver20@naver.com (B.-E.J.); ckstjd5091@naver.com (C.-S.K.); dlwldms4535@naver.com (J.-E.L.); jhcha@pusan.ac.kr (J.C.)
[2] Microbiological Resource Research Institute, Pusan National University, Busan 46241, Republic of Korea; moonko81@nate.com (K.M.)
[3] Department of Biological Sciences, Pusan National University, Busan 46241, Republic of Korea
[4] Department of Asian Food and Culinary Arts, Youngsan University, Busan 48015, Republic of Korea; inmpark@gmail.com
[5] Department of Biological Sciences, Southern Methodist University, Dallas, TX 75206, the United States of America; kohs@mail.smu.edu
[6] Division of Hepatobiliary and Pancreas Surgery, Department of Surgery, Biomedical Research Institute, Pusan National University, Busan, 46241, Republic of Korea; ymh@pusan.ac.kr
* Correspondence: kimjn@pusan.ac.kr (J.N.K.); Tel.: +82-51-510-2269, kimsw@pusan.ac.kr (S.-W.K.); Tel.: +82-51-510-2260

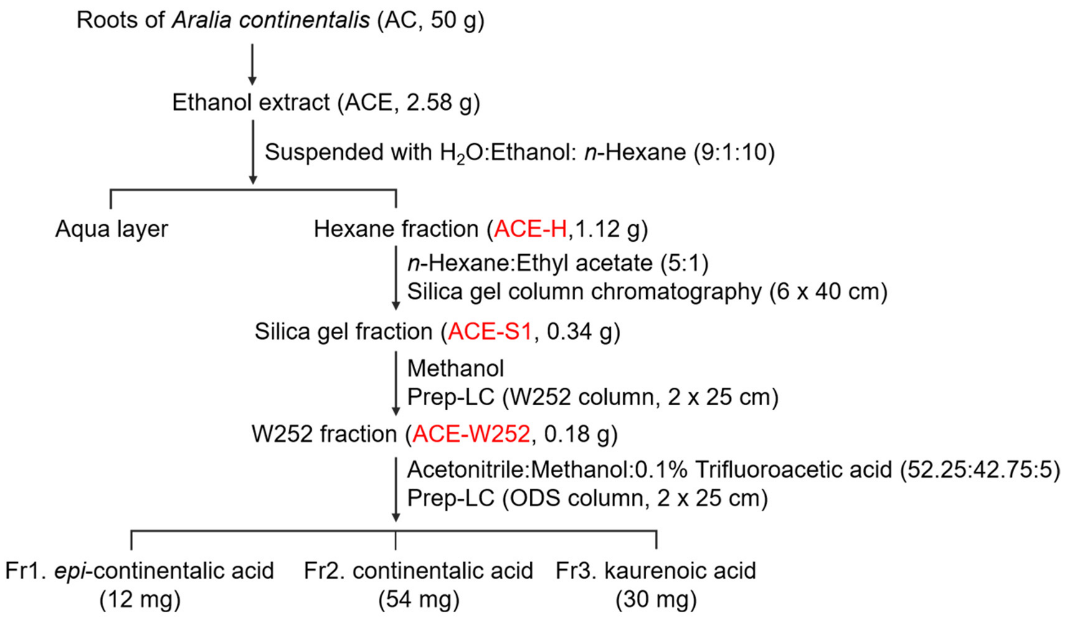

Figure S1. Extraction process of diterpenoids from the roots of *Aralia continentalis*.

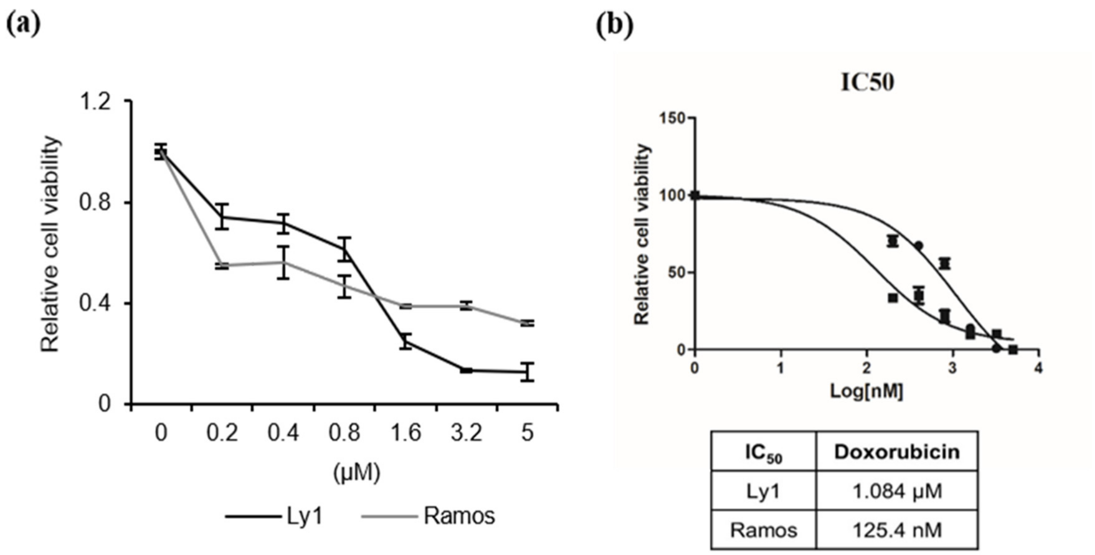

IC_{50}	Doxorubicin
Ly1	1.084 μM
Ramos	125.4 nM

Figure S2. The anti-lymphoma effect of doxorubicin. (**a**) Ly1 and Ramos cells were treated with Doxorubicin (0, 0.2, 0.4, 0.8, 1.6, 3.2, 5 μM) for 24 h, and cell viability was measured using MTS assay. (**b**) The IC_{50} values of Doxorubicin in Ly1 and Ramos cells were calculated using GraphPad Prism 5 software.
